# Prioritising quality: investigating the influence of image quality on forensic facial comparison

**DOI:** 10.1007/s00414-024-03190-7

**Published:** 2024-02-22

**Authors:** Nicholas Bacci, Nanette Briers, Maryna Steyn

**Affiliations:** 1 Human Variation and Identification Research Unit, School of Anatomical Sciences, Faculty of Health Sciences, University of the Witwatersrand, Johannesburg, South Africa; 2 Division of Clinical Anatomy, Faculty of Medicine and Health Sciences, University of Stellenbosch, Stellenbosch, South Africa

**Keywords:** Facial comparison, Facial identification, Image quality, Resolution, Lighting, Morphological analysis

## Abstract

Morphological analysis in forensic facial comparison was recently validated for judicial use. However, no image quality assurance systems exist for this purpose, despite image triage being considered the best practice approach. Hence, this study aimed at testing a semi-quantitative scoring method to assess image quality and investigated facial image resolution and lighting quality quantitatively in a context of forensic facial comparison. For this purpose, 400 facial comparison photographic and CCTV image pools developed from the Wits Face Database were used. These facial images were analysed in prior studies that investigated the validity of morphological analysis. A semi-quantitative image quality scoring system was adapted and tested on the above sample and compared across correct and incorrect matches obtained as part of previous studies using a logistic regression model. In addition, facial images were cropped to the closest pixel comprising the face, head and neck areas; then, a face-to-image pixel proportion was calculated as an estimator of resolution quality; and pixel exposure qualities were obtained to be compared to facial comparison outcomes. Ideal and high image quality scores were related to correctness of matches, while low-quality scores were related to incorrect matches. High pixel proportions were related to true matches and low exposure was related to false positives, while high exposure was related to false negatives. These results suggest that an easy method for image triage could be employed by scoring image quality. Quantitative measures should be investigated further for thresholding quality suitability for confidence of facial comparisons.

**Supplementary information:**

The online version contains supplementary material available at 10.1007/s00414-024-03190-7.

## Introduction

Facial identification has received considerable attention in recent years, both in its semi-automated form of facial recognition systems and in its human expert-based form of forensic facial comparison (FFC). The most notable concern regarding the performance of facial comparison, and a topic for consideration in facial recognition as well, is image quality. Obtaining images of high quality has been considered a priority for both these approaches to facial identification, with reduced accuracies reported both in facial comparison [1, 2] and facial recognition technology [3].

Image quality deficiencies have been considered by many as a concern that can reduce face matching ability [1, 4–10]. Factors causing reduced image quality include camera specifications, camera placement, lighting conditions and data loss or corruption. Each of these factors leads to specific effects on the images to be analysed, ranging from low-resolution images with a decrease in facial detail, artefacts and image distortion that reduce usable detail, shadowy and dark areas that alter facial appearance and low clarity of images in general [2]. Some of the principal factors receiving the greatest focus have been pixel count, facial image resolution [1, 9] and face exposure conditions [1, 11].

Recently, several algorithms have been developed to assess image quality for facial recognition systems, with a recent study contributing significantly to this affect [3]. However, these approaches to assess image quality have not been tested in human-based facial comparisons and are often limited to the conditions set up by studies for specific environments. The aim of this study was to evaluate the effect of the quality of images used for facial comparison on comparison outcomes. For this purpose, an ordinal method of scoring image quality was adapted from previously published work [4] in conjunction with alternative methods to quantify image qualities, by investigating face resolution as well as under- and overexposure.

## Materials and methods

The sample for the present study included all facial comparisons previously conducted as part of a series of tests validating FFC using the Facial Identification Scientific Working Group (FISWG) guidelines [1, 12, 13]. No new comparisons were performed for the purposes of this study. These comparisons included a total of 400 face pools, each composed of one target image from one of various photographic or CCTV conditions and 10 potential matching photographs captured under standardised conditions (Table 1). All images were obtained from the Wits Face Database [14, 15].

**Table 1:** Outcomes of facial comparisons by face pool cohort [1, 12]

Face pool cohort	True positives	True negatives	False positives	False negatives	Inconclusive analysis (excluded)	Totals
Wildtype photographs	69 (92.0%)	5 (6.7%)	0 (0.0%)	1 (1.3%)	0 (0.0%)	**75**
Standard CCTV images	71 (71.0%)	3 (3.0%)	21 (21.0%)	2 (2.0%)	3 (3.0%)	**100**
Eye-level CCTV images	87 (91.6%)	5 (5.2%)	1 (1.1%)	2 (2.1%)	0 (0.0%)	**95**
Analogue CCTV images	27 (20.8%)	6 (4.6%)	2 (1.5%)	85 (65.4%)	10 (7.7%)	**130**
**Totals**	**254**	**19**	**24**	**90**	**13**	**400**

In the original studies [1, 12, 13], a total of 13 face pools, primarily from the analogue and standard CCTV cohort, were excluded during morphological analysis due to particularly poor image conditions which made comparison impossible. A total of 387 face pools were thus analysed from which true positive, true negative, false positive or false negative outcomes were obtained as shown in Table 1. Face pools that obtained an inconclusive analysis score (thereby initially being excluded) during comparison were still included in analyses when examining comparison outcomes (face-to-image pixel proportions and lighting quality). However, these face pools marked as inclusive analyses were excluded when correct and incorrect matches were considered only (compared to image quality scoring).

All target images were scored for quality, while all the database standardised images were considered to have the highest quality level as they were controlled and intended to be ideal standardised images for comparison. Target images were scored using an adaptation of Schüler and Obertová’s image quality scoring criteria [4] (Table 2). The scoring system adaptation involved the inclusion of specific facial comparison best practice guidelines (according to FISWG). For this inclusion, the concept of the percentage of the face that is usable in a facial comparison was expanded to the facial components FISWIG recommends [16]. Specifically, reference was made to the standard 15 facial components each face is expected to have. Facial components 16 (scars), 17 (facial marks, such as moles, discolouration etc.) and 18 (alterations, such as tattoos and piercings) were excluded from this scoring system as these are not necessarily present. Facial components were considered unusable if no descriptors could be used from the FISWG feature list to describe the feature. If even a superficial description could be made of a facial component, it was considered usable. Bilateral features were considered together, meaning that if one ear, for example, was adequately visible, it was considered usable for facial comparison. However, in cases where the light or other factors might mislead the assessment of facial features on one side of the face compared to the other, it was considered limiting the utility of that feature or set of features. To assess intra- and inter-observer rater agreement, a sub-set of 16 original target images were re-scored by the author and by an observer trained in the scoring criteria.

**Table 2:** Scoring criteria for image quality evaluation adapted from Schüler and Obertová [4]

Quality level	Quality score name	Definition
1	Optimal	The quality of the image corresponds particularly well to the requirements of morphological analysis. The resolution, sharpness and illumination of the image are excellent. The face, head and neck areas are not obscured, and image artefacts are not present. Small-scale facial features and skin structures (i.e. texture, luminance, fine hair) are visible and can be described in detail
2	Good	The quality of the image fully meets the requirements of morphological analysis. Resolution, sharpness and illumination are good. The percentage of object overlay (obstructions/disguises) and image artefacts is a maximum of 5% of the face, head and neck areas, indicating a maximum of 1 facial component from FISWG not being usable in the morphological process. The small-scale facial features and skin structures (i.e. texture, luminance, fine hair) are visible but details are at least somewhat unclear
3	Satisfactory	The quality of the image is sufficient to meet the requirements of morphological analysis. A few deficits can be detected in the resolution, sharpness or illumination. The percentage of object overlay or image artefacts is greater than 5% but smaller than 30% of the face, head and neck areas; effectively, between 2 and 4 facial components from FISWG are not usable. Small-scale features can be described; however, skin structures are no longer visible
4	Sufficient	The quality of the image is sufficient to meet the requirements of morphological analysis. The percentage of object overlay and image artefacts is greater than 30% but smaller than 65% of the face, head and neck areas; between 5 and 9 facial components from FISWG are not usable. Small-scale features can be described either to a very limited extent or not at all
5	Poor	The quality of the image still meets some requirements for morphological analysis, with some clear deficiencies or limitations; an evaluation is hardly possible without optimised comparative images (i.e. matching conditions of image capture, such as angle and facial expression). The resolution, sharpness and illumination show clear deficiencies. The percentage of the face, head and neck areas with overlay and image artefacts is more than 65% but less than 80%, meaning that between 10 and 12 facial components from FISWG are not usable. Only large-scale features (e.g. overall nose or ear shape and relative size, cranial vault/head shape) can be described confidently
6	Insufficient	The quality of the image is completely insufficient for morphological analysis. The resolution, sharpness and illumination are deficient and create artefactual modifications or an obscuring overlay over 80% of the craniofacial morphology, effectively over 12 facial components from FISWG not being usable. The colour depth of the image may also be insufficient (less than 8 bit). The number of describable features is insufficient for an evaluation

In addition to the image quality scoring described in Table 2, for each facial image, the width and height in pixels that made up the face, head and neck areas were recorded in an effort to determine whether there is a relationship with actual pixel counts and FFC accuracy. This was performed by cropping the target images down to the last visible pixel contributing to the face, neck and head region using Microsoft Paint, an easy-to-use and accessible tool to do this by anyone with a simple Windows computer, and then capturing the actual pixel dimensions from the now cropped target image of the face alone. Pixel dimensions were captured separately and after the individual images were scored on the quality classification scheme. No other image parameters were altered during pixel dimensions capture, including the colour bit depth and the density of pixels per inch (dpi). The same 16 original target images used for intra- and inter-observer agreement testing were also cropped a second time by the author and an additional observer following the above procedure.

The proportion of these new cropped face target images over the overall original target image total pixels was computed. This calculation indicated the number of pixels that the face was roughly comprised of, regardless of original image resolution. As this was not considered a universal indicator of facial image quality, it was termed as “face-to-image pixel proportion” or FIPP.

Lighting information was extracted from each cropped face image’s histogram using Fiji (version 2.13.1) [17]. The data extracted as part of this set of analyses were referred to as face exposure. To do this automatically, two different ImageJ macro scripts were developed. One script batch processed all cropped facial images in a folder and recorded the total number of pixels and the counts of pixels under each greyscale value at the 256 bins generated by Fiji (ImageJ Macro Code S1). A second script recorded the mean, mode, median, standard deviation, skewness, minimum and maximum greyscale value for each histogram (ImageJ Macro Code S2). These macros were developed using the Fiji functions and the “Read and Write Excel” plugin originally developed by Anthony Sinadino (http://imagej.net/User:ResultsToExcel) and then updated by Brenden Kromhout (https://github.com/bkromhout/Read_and_Write_Excel_Modified).

**Fig. 1:**
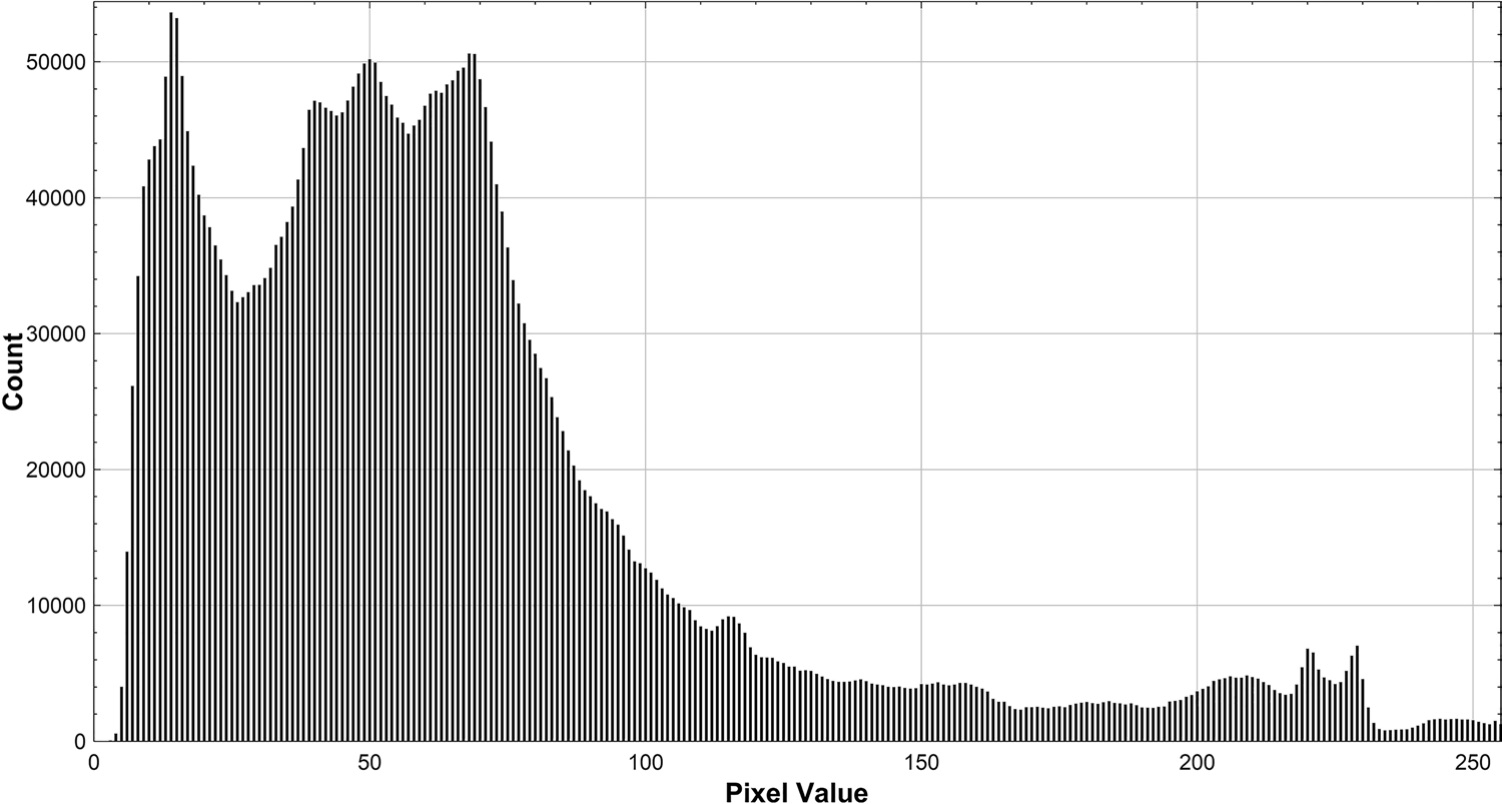
Greyscale histogram example from a target wildtype cropped facial photograph inclusive primarily of the face. Pixel value bins represent the greyscale value of individual pixels, with 0 being completely black and 255 completely white. High-resolution histogram obtained using the HistogramPlotter Fiji plugin (https://imagej.nih.gov/ij/macros/HistogramPlotter.txt)

An example of these histograms is shown in Fig. 1. The data extracted from each facial cropped image from these histograms included total pixels, pixel counts at each of the 256 greyscale values of the histogram, mode greyscale value, mean greyscale value, median greyscale value, skewness of the histogram and standard deviation. In addition to the manual cropping of images, the total count of pixels in each face cropped image as well as the face-to-image pixel proportion was recorded. From the greyscale pixel value dataset, four variables were considered:

1. Underexposed face ratio (UFR), quantified as the sum of greyscale values in the first 10 darkest bins of the histogram divided by the overall facial image pixel count
2. Overexposed face ratio (OFR), quantified as the sum of greyscale values in the last 10 brightest bins of the histogram divided by the overall facial image pixel count
3. Facial image greyscale value mean, suggestive of the overall darkness or brightness of the image
4. Facial image greyscale histogram skewness as an indicator of the brightness in a given image

All statistical analyses were conducted in RStudio (v. 4.3.0) [18]. The following plugins were used: tidyverse [19], ggplot2 [20] and ggpubr [21]. An alpha value of *p* < 0.05 was considered significant. Summary statistics were computed using the base R packages and graphs were made using ggplot2 [20]. Three main grouping variables were used for comparison purposes: image type, comparison outcome and match correctness. Image types included the four types of images recorded: wildtype photographs, standard CCTV, eye-level CCTV and analogue CCTV. Comparison outcomes included the final outcomes of each face pool comparison, namely true positive, true negative, false positive, true negative and inconclusive analysis. Match correctness was intended to combine the comparison outcomes into a binary metric, as such correct matches were considered for any face pool that obtained a true positive or true negative match. Incorrect matches were considered any face pool that obtained a false positive or false negative match, while inconclusive analyses were excluded from this group of analyses. Intra- and inter-observer errors for the quality scoring system were computed using a quadratically weighted Cohen’s kappa using the irr package [22], and for the pixel totals following cropping of facial images, a two-way, mixed, single-measures intraclass correlation coefficient using the psych package [23].

A logistic regression model was computed to identify the ability of the image quality scores to predict whether matches would be correct or incorrect (match correctness). The logistic regression was also hyperparameter tuned to obtain the highest possible predictive power using glmnet [24] and tidymodels [25] packages for RStudio. The sample was randomly split as part of the logistic regression testing, with 80% of the sample being used to train the model and the remaining 20% used to test the model. The logistic regression prediction coefficients were plotted against the scores using ggpubr [21]. The accuracy of the logistic regression model was also computed, as were the recall (Eq. 1) and precision (Eq. 2) for the logistic regression model.

1 $$\mathrm{Recall}:\frac{\mathrm{True}\;\mathrm{Positives}\;\mathrm{of}\;\mathrm{Logistic}\;\mathrm{Regression}}{\mathrm{True}\;\mathrm{Positives}+\mathrm{False}\;\mathrm{Negatives}\;\mathrm{of}\;\mathrm{Logistic}\;\mathrm{Regression}}$$

2 $$\mathrm{Precision}:\frac{\mathrm{True}\;\mathrm{Positives}\;\mathrm{of}\;\mathrm{Logistic}\;\mathrm{Regression}}{\mathrm{True}\;\mathrm{Positives}+\mathrm{False}\;\mathrm{Positives}\;\mathrm{of}\;\mathrm{Logistic}\;\mathrm{Regression}}$$

Comparisons between face-to-image pixel proportions and image type, comparison outcomes and match correctness were conducted with Kruskal-Wallis ANOVA by ranks test as the data were not parametric. *Post hoc* testing was conducted using pairwise Wilcoxon rank sum tests with a Bonferroni correction.

## Results

### Semi-qualitative scoring of image quality

The newly adapted image quality scoring suggests a vastly different picture of which images were suitable for comparison than previously considered on a completely qualitative basis during the original analysis of these face pools in prior studies. Based on this new image quality scoring approach, the majority of wildtype photographs and eye-level CCTV images were considered to be of ideal to appropriate quality for comparison. Standard CCTV images showed a fluctuating quality score, but all on the lower end of the quality scoring system. Concerningly, all analogue data were scored as 6, indicating the lowest quality that is not at all suitable for facial comparison (Table 3). Intra- and inter-observer agreement for quality scoring showed a perfect (*κ* = 1.00, *p* = < 0.001) and nearly perfect (*κ* = 0.945, *p* = < 0.001) agreement, respectively.

**Table 3:** Quality scores across face pool cohorts. For quality score definitions, refer to Table 2

Face pool cohort	Score 1	Score 2	Score 3	Score 4	Score 5	Score 6
Wildtype photographs	31	34	10	0	0	0
Standard CCTV	0	0	15	43	17	25
Eye-level CCTV	36	38	19	2	0	0
Analogue CCTV	0	0	0	0	0	130
**Totals**	**67**	**72**	**44**	**45**	**17**	**155**

**Fig. 2:**
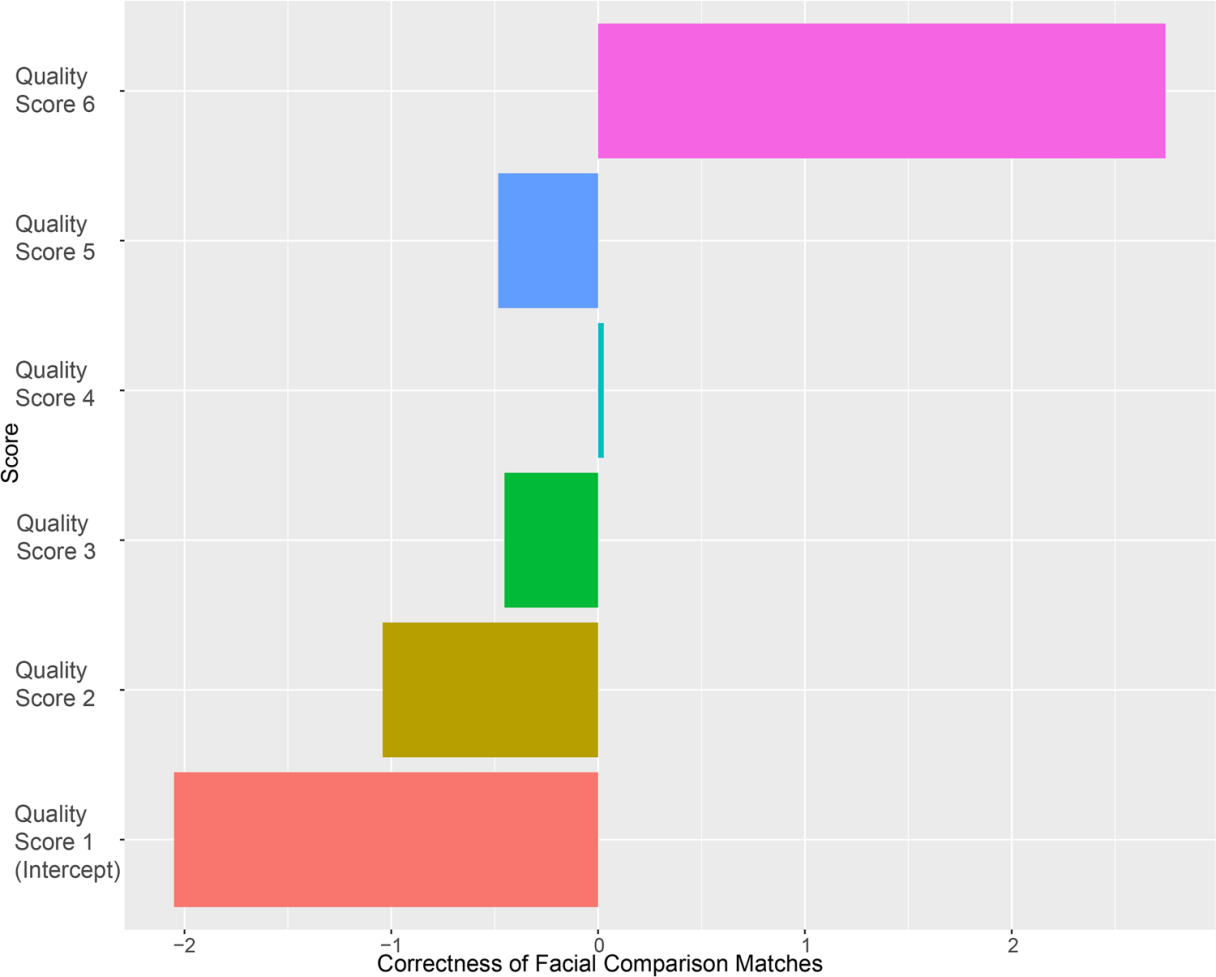
Graph of logistic regression model coefficients used to predict correctness of facial comparison matches by image quality scores. Negative coefficients (*x* axis) represent increased probability of estimating a correct match for each score (*y* axis) and positive coefficients the probability of estimating incorrect matches. Coefficients with a value greater than 0.5 (quality scores 1, 2 and 6) predict correctness of facial comparison matches better than the others (quality scores 3, 4 and 5). Generated in R studio using ggplot2 [20]

When investigating image quality scoring (Table 2) in relation to match correctness, the logistic regression model demonstrated a strong ability of the highest and lowest quality scores to predict correct and incorrect matching ability, respectively (Fig. 2; Tables 4 and 5). The accuracy obtained for this model was 85.9%, with a precision of 95.8% and a recall of 83.6%. Overall, estimate coefficients (Table 5) aligned relatively well with facial comparison match correctness, apart from scores of 4 and 5. When only estimate coefficients greater than 0.5 were taken into consideration, image quality scores of 6, mostly attributed to standard CCTV and analogue CCTV images, were a good indicator of obtaining incorrect matches; image quality scores of 1 and 2 were good indicators of obtaining correct matches (Fig. 2). Scores of 4 and 5, not being representative of match correctness, potentially suggested a suspicion that arose during quality data collection, namely that quality alone is not enough of an indicator for match correctness.

**Table 4:** Confusion matrix of logistic regression model in the sub-sample^a^ proportion used for testing of the model

Prediction	Actual	
	Correct	Incorrect
Correct	46	2
Incorrect	9	21

^a^The sub-sample included 20% of the 387 target face images (*n* = 78) as a sub-set for testing the model that was trained on the other 80% of target images (*n* = 309)

**Table 5:** Logistic regression model coefficients when predicting correctness of facial comparison matches by image quality scores, with the associated adjusted penalty values^a^ allocated to the coefficients after training the model

Score	Estimate coefficient	Coefficient penalty
Quality score of 6	2.740	1 × 10^−10^
Quality score of 5	–0.453	1 × 10^−^^10^
Quality score of 4	0.0277	1 × 10^−^^10^
Quality score of 3	−0.483	1 × 10^−^^10^
Quality score of 2	−1.040	1 × 10^−^^10^
Intercept (score 1)	−2.050	1 × 10^−^^10^

^a^The penalty values were used to reduce the coefficients’ effects on the model based on their contribution to obtain the optimal logistic regression model, to prevent overfitting the model

### Quantification of image quality

#### Face resolution

Face-to-image pixel proportion (FIPP), the number of pixels that made up a face in each image divided by the total number of pixels of the image, was closely aligned to the image type (Table 6). The more detailed faces were captured by the wildtype photographs and eye-level CCTV (Table 6), as the face was either close or the camera had a narrower field of view than the ceiling-mounted standard and analogue CCTV cameras. The lighting data showed some consistency of light exposure with image type as well, with standard CCTV having some of the darkest pixel greyscale value ratios, and the least light pixel greyscale values on average as well as the lowest mode indicating overall a larger number of pixels closer to black greyscale values (Table 7). The other image types had more variety with more exposed images in both wildtype photographs and analogue images (Table 7). However, analogue CCTV images also showed the least positive skewness in their histograms, indicating overall less outliers of greyscale pixel values (Table 7). The intra- (ICC = 1, *F* = 8508, df = 15, *p* < 0.001, lower and upper bound of 1) and inter-observer agreement (ICC = 1, *F* = 5856, df = 15, *p* < 0.001, lower and upper bound of 1) was perfect for the cropped images’ total pixel values.

**Table 6:** Descriptive statistics for quality score and face-to-image pixel proportion

Face pool cohort	Median quality score	Mean FIPP^a^	FIPP^a^ standard deviation	FIPP^a^ coefficient of variation
Wildtype photographs	2	27.64%	3.80%	0.14
Standard CCTV	4	0.46%	0.09%	0.19
Eye-level CCTV	2	14.41%	4.40%	0.31
Analogue CCTV	6	0.41%	0.06%	0.14

^a^*FIPP* face-to-image pixel proportion

**Table 7:** Descriptive statistics for face exposure across the various image modalities

Face pool cohort	Mean black bin ratio	Mean white bin ratio	Mean mode pixel greyscale value	Mean pixel value skewness
Wildtype photographs	8.04%	2.87%	86.83	1.04
Standard CCTV	32.08%	0.75%	21.20	1.61
Eye-level CCTV	3.94%	1.79%	49.88	1.90
Analogue CCTV	4.39%	2.44%	84.85	0.65

Associations between face-to-image pixel proportions (FIPP) and imaging modalities, comparison outcomes and match correctness were identified. A significant difference was found between face-to-image pixel proportions across the various imaging modalities (KWA chi-squared = 319.69, df = 3, *p*-value < 0.001), and this difference was significant between every imaging modality (Table 8). In descending order, images acquired from each of the following set-ups had less pixels of the image dedicated to the face: wildtype photographs (WT photos), eye-level closed-circuit television (EL CCTV), standard closed-circuit television (ST CCTV) and analogue closed-circuit television (AL CCTV) (Supplementary Information: Fig. 1). Significant differences were also seen across comparison outcomes when FIPP was considered (KWA chi-squared = 133.11, df = 4, *p*-value < 0.001), specifically between true positives and negatives, and all other comparison outcomes, excluding each other (Table 9). Barring a few comparisons, the majority of images with FIPPs above 6% resulted in true positives or true negatives, despite almost a quarter of the sample (split between AL CCTV and ST CCTV exclusively) having true positive and true negative outcomes (Supplementary Information: Fig. 2).

**Table 8:** Pairwise comparison using Wilcoxon rank sum test with Bonferroni continuity correction between image types and face-to-image pixel proportion

	Analogue CCTV	Eye-level CCTV	Standard CCTV
Eye-level CCTV	< 0.001*	-	-
Standard CCTV	< 0.001*	< 0.001*	-
Wildtype photographs	< 0.001*	< 0.001*	< 0.001*

*These values indicate significant differences between image modality cohorts

**Table 9:** Pairwise comparisons using Wilcoxon rank sum test with Bonferroni continuity correction between comparison outcomes and face-to-image pixel proportion

	False negative	False positive	Inconclusive analysis	True negative
False positive	1.000	-	-	-
Inconclusive analysis	1.000	1.000	-	-
True negative	< 0.001*	= 0.010*	= 0.011*	-
True positive	< 0.001*	< 0.001*	< 0.001*	1.000

*These values indicate statistically significant differences in face-to-image pixel proportion between two categories of outcomes

#### Face lighting quality and exposure

Lighting quality, interpreted as under- and overexposure of the faces, was tested for any association with facial comparison accuracy. When comparing the sum of the first 10 darkest bins of the greyscale pixel values to facial comparison outcomes, significant differences were not only seen between true outcomes (positives and negatives), and all false negatives and inconclusive analyses, but also between false positives, and false negatives and inconclusive analyses (KWA chi-squared = 149.5, df = 4, *p*-value < 0.001) (Supplementary Information: Table 1). This analysis indicated that when more underexposed pixels were present, matches tended to be true positives, true negatives and false positives rather than false negatives and inconclusive analyses. When comparing the sum of the last 10 brightest bins of the greyscale pixel values to comparison outcomes, significant differences were identified between true outcomes (positives and negatives), and false outcomes and inconclusive analyses (KWA chi-squared = 73.178, df = 4, *p*-value < 0.001) (Supplementary Information: Table 2). This result indicated that when more overexposed pixels are present, a greater propensity for true matches than false and inconclusive analyses was observed. However, these total numbers of pixels that are under- and overexposed are not necessarily representative of the image exposure quality, as smaller images will have less pixels of either dark or bright greyscale values, potentially hiding the effect that appropriate exposure plays in facial comparison; as such, proportions were also compared.

 When considering underexposed face pixel ratios (UFR) and overexposed face pixel ratios (OFR), a slightly different picture emerged. Comparison outcomes were significantly different for the under- and overexposed pixel ratios (UFR: KWA chi-squared = 24.919, df = 4, *p*-value < 0.001; OFR: KWA chi-squared = 33.581, df = 4, *p*-value < 0.001). When a false positive outcome was obtained, a high proportion of underexposed pixels was seen significantly more often than with other outcomes (Table 10, Supplementary Information: Fig. 3). Similarly, a significantly higher proportion of overexposed pixels was seen when false positives occurred, than with other outcomes, excluding inconclusive analyses (Table 11, Supplementary Information: Fig. 4). These results suggest that underexposure played a larger role than overexposure in the occurrence of false positives, but not with other outcomes. Interestingly, with false negative outcomes, a significantly higher overexposure was also seen when compared to true positives and false positives than with other outcomes (Table 11, Supplementary Information: Fig. 4).

**Table 10:** Pairwise comparisons using Wilcoxon rank sum test with Bonferroni continuity correction between comparison outcomes for underexposed pixel ratio

	False negative	False positive	Inconclusive analysis	True negative
False positive	< 0.001*	-	-	-
Inconclusive analysis	1.000	0.038*	-	-
True negative	0.999	0.005*	1.000	-
True positive	1.000	< 0.001*	1.000	1.000

*These values indicate significant differences between two categories of outcomes

**Table 11:** Pairwise comparisons using Wilcoxon rank sum test with Bonferroni continuity correction between comparison outcomes for overexposed pixel ratio

	False negative	False positive	Inconclusive analysis	True negative
False positive	< 0.001*	-	-	-
Inconclusive analysis	0.196	1.000	-	-
True negative	1.000	0.003*	0.474	-
True positive	0.003*	0.005*	1.000	1.000

*These values indicate significant differences between two categories of outcomes

 When the mean greyscale pixel value (MGV) was considered, significant differences were seen between true outcomes (positive and negative) and false outcomes (positive and negative), as well as between false positives and negatives (KWA chi-squared = 118.84, df = 4, *p*-value < 0.001) (Table 12, Supplementary Information: Fig. 5). False positive outcomes were significantly associated with lower MGVs compared to other outcomes, and false negative outcomes were significantly associated with slightly increased MGVs (Table 12, Supplementary Information: Fig. 5).

**Table 12:** Pairwise comparisons using Wilcoxon rank sum test with Bonferroni continuity correction between comparison outcomes for mean of greyscale pixel values

	False negative	False positive	Inconclusive analysis	True negative
False positive	< 0.001*	-	-	-
Inconclusive analysis	1.000	0.021*	-	-
True negative	0.020*	< 0.001*	1.000	-
True positive	< 0.001*	< 0.001*	0.308	0.290

*These values indicate significant differences between two categories of outcomes

 Greyscale histogram skewness (GHS) compared to facial comparison outcomes appeared to distinguish better between false negatives and other outcomes and between false positives and inconclusive analyses (KWA chi-squared = 92.69, df = 4, p-value < 0.001) (Table 13, Supplementary Information: Fig. 6). This suggests that when outcomes were false negatives, the greyscale values were overall brighter (less skewed histograms with relatively more evenly distributed pixel counts across greyscale values) (Table 13, Supplementary Information: Fig. 6).

**Table 13:** Pairwise comparisons using Wilcoxon rank sum test with Bonferroni continuity correction between comparison outcomes for skewness of greyscale pixel value histograms

	False negative	False positive	Inconclusive analysis	True negative
False positive	< 0.001*	-	-	-
Inconclusive analysis	0.692	0.021*	-	-
True negative	0.002*	0.195	1.000	-
True positive	< 0.001*	0.439	0.127	1.000

*These values indicate significant differences between two categories of outcomes

## Discussion

Image quality is a crucial component of any image analysis or comparison discipline. In facial comparison, it is recommended to triage images before attempting morphological analysis [26]. This can be of benefit to both avoid human resource mismanagement, but also to limit instances of inaccurate matches, particularly in a forensic setting where false positives could lead to false imprisonment. The importance of image quality in these disciplines, and specifically facial comparison, not only makes investigating its relationship with the performance of comparison methods vital to optimise image triage, but also elucidates its performance and defensibility in a court of law. The effect of image quality is often assumed, but not quantifiably demonstrated. In addition, a simple manner to assess this aspect without using convoluted neural networks, for example [3], not tested in human-based facial comparison applications, was missing. This research aimed to address this issue.

### Image quality scoring

The original scoring system [4] is a generic approach to image quality with rough percentages used to estimate the amount of detail visible in a face. The FISWG feature list is the only validated [12] and considered best practice approach to facial comparison [16, 27]; as such, the facial components from the FISWG feature list were at this point incorporated into the scoring system (Table 2) in an attempt to semi-quantify the quality scoring approach. Scoring image quality with this adapted method proved simpler and more straightforward than attempting to estimate percentage of the face usable, as seen by the high intra- and inter-observer agreement. Overall, this image quality scoring method provided an effective approach to estimate effectiveness of applying facial comparison successfully. A score of 6, defined as an image quality completely insufficient for morphological analysis, was indeed particularly well aligned with incorrect matches (Fig. 2). Scores of 1 and 2, defined as ideal for and meeting the requirements for morphological analysis, respectively, were good indicators of correct match outcomes (Fig. 2). The prediction ability of an image quality scoring system like this is a vital starting point for being able to determine the ability of analysts to obtain correct matches. Eliminating images that would be more likely to result in an incorrect match with the use of a large number of analyst labour hours, in already constrained forensic settings, is a worthwhile pursuit.

Visual grading analysis of images has been conceptually considered before in medical imaging [28]. This has often been countered by arguments suggesting it can become a “beauty-contest” of data that is not scientifically validated [29]. The current study has, however, used a logistic regression model to demonstrate that this adapted quality scoring method has good predictive power to assign correct facial comparison matches to higher quality images and vice versa. This method follows a similar approach to what is recommended for both for radiological imaging [28, 30] and facial comparison [4, 26]. It is vital for a grader to outline their ability to fulfil the requirements of facial comparison by morphological analysis based on the images’ quality. To this end, the findings of the current study are not only indicative of a good predictive capacity of the scoring system, but also demonstrates that in previous studies that used this same dataset of facial comparisons, many of the lower quality analogue CCTV images and some standard CCTV images with artefacts [1, 12] should probably have been excluded from analysis and never compared. The confirmation of needing to exclude these sub-optimal images is a highly valuable insight as these low-quality images were included in some of the face pool cohorts with lower chance corrected accuracies across all the above validation studies [2]. This insight would facilitate abiding by the principle of presumption of innocence, which most territories recognised by the United Nations followed by reducing the risk of false positive facial comparison outcomes. Sub-optimal images, however, may still be used for facial review and facial assessment in the investigative phases of a case, as they could still be of value if stronger, more definitive evidence can be gathered in that process with the aid of these poor-quality images. Avoiding the usage poor-quality images in the facial examination processes can also aid in severe case-load reduction for facial comparison analysts [30], especially in resource-constrained settings.

One notable limitation of this scoring system is that it does not take into consideration which facial features are affected by poor-quality images other than the fine detail (e.g. skin texture, fine hair), and at the current stage, it is suspected that certain facial features would be more useful in morphological analysis than others, but this has not been statistically demonstrated yet. In our prior validation studies, we qualitatively suggested that contrary to popular belief, the eyes were not of benefit to most analyses, while the ear and nose seemed to aid during the facial comparison the most [1, 2, 12, 13]. The importance of ear morphology is corroborated by other studies [31] that have found the ear to be a unique feature of the face [32, 33]. However, the specific benefits of visibility of individual features in facial comparison need to be statistically demonstrated prior to considering adjusting the current scoring system to incorporate some form of weighting of the quality scoring based on the most helpful facial features. Despite this, a preliminary image quality scoring system such as the one tested in the present study remains of high value as it was repeatable and easy to apply and still yielded valuable information about the value of the image data available for a facial comparison analysis. To this end, certain government identification agencies have instituted considerations of image quality in their facial comparison practices. For example, the Danish National ID Centre that performs facial and fingerprint comparisons has incorporated an image quality assessment phase in their reports that informs the weight of potential facial comparison matches (and non-matches) using a decision tree approach (personal communication from Stine Nordbjærg and Trine Arp Edvardsen from the Danish National ID Centre). While this approach has yet to be tested extensively, this kind of implementation is crucial for successful and efficient application of facial comparison, which can reduce an analyst’s confirmation bias and resource costs.

### Quantification of image quality

#### Face resolution

Despite the benefit of a scoring system to guide image triage, more in-depth, technical considerations of image quality may aid in understanding the specific occurrence of false positives and false negatives across various circumstances. Further research quantifying image quality in facial comparison contexts could potentially lead to identifying thresholds for image quality to either associate a particular comparison with a “level of confidence” or exclude inadequate images from further facial examination.

In the current study, when attempting to quantify specific image quality indicators, face-to-image pixel proportion (as an indicator of face resolution) and greyscale values (as an indicator of face exposure levels) were shown to have clear relationships to the facial comparison outcomes. Associations between imaging modality were evident with notably higher face-to-image pixel proportion (FIPP) seen across WT photos and EL CCTV compared to ST and AL CCTV. Higher FIPPs (greater than 6%) were also more often seen with true positive and true negative outcomes, which is indicative of how a greater face resolution, irrespective of original image resolution, would provide more visible detail and information allowing for a more successful comparison outcome. The clear, high resolution of faces in wildtype photographs and eye-level CCTV images resulted from not only the overall high resolution of the WT photos (18 MP, 5 m distance), but also the proximity of the face to the EL CCTV camera (4MP, 0.8-m distance). The importance of camera to subject distance is evidenced by the fact that the ST CCTV camera was identical to the EL CCTV, yet the distance from the camera and the wide field of view reduced the FIPPs and overall number of facial components clearly visible to that camera. The effect of distance on image quality is a further indicator of the importance of camera placement.

The relevance of camera placement has been suggested previously in relation to the angle of incidence imposed on the face [34–36], yet distance to subject and field of view have not been investigated in consideration of subject resolution. This is an expected downside of placing CCTV installations at ceiling height positions (usually around 2.5 and 3 m above ground), intended to have a wide surveillance range for reduced cost [36, 37]. However, it comes at a great loss of image detail, even when the camera has the same specifications. As shown in the current study, this loss of image detail can severely hinder facial comparison capabilities. Placing additional cameras at eye-level in high-risk locations of a monitored area, for the specific intention of acquiring optimal images for facial comparison, would likely greatly aid in optimal facial image quality acquisition. Mounting cameras at better suited heights and locations can also be a potential benefit to both the investigation processes, where clearer view of any illicit activities may be captured (e.g. drug transactions), and any other image comparison techniques that would require better quality images, such as hand and car comparisons.

Resolution has been considered a primary factor of image quality across the literature (e.g. [1, 5, 9, 10]). In the current study, the vast majority of FIPPs above 6%, primarily belonging to WT photos and EL CCTV, yielded only one false positive and three false negatives, without any exclusions. Most false positives (23), false negatives (87) and all exclusions (13) occurred with FIPPs below 1%, found in ST CCTV and AL CCTV images. This suggests that a threshold for minimum face-to-image resolution may be a particularly good indicator for image quality; however, further testing with FIPPs between 6% and less than 1% is required to identify the ideal threshold where the proportion of incorrect matches exceeds correct matches by more than 20%, as general acceptance of validity for forensic techniques is 80% reproducibility (e.g. from fingerprint analysis [38]).

The predisposition of quantified face resolutions to specifically either false positives or negatives has not been discussed previously in relation to minimum facial image resolution requirements. One exception to this was described by Damjanovski [9], who emphasises the size requirements of the face in each image to allow facial examination. Specifically, Damjanovski [9] recommends, based on the ISO-62676 recommendations, that a target needs to represent a minimum of 400% of the screen height (minimum of 1 mm per pixel; effectively, the original recorded image represents approximately one-quarter of the whole individual, including the face). While these pixel measurements were not available for the current sample, due to the original study design, it would be evident that images with face-to-image pixel proportions below 6% would not represent enough of the face to be suitable for comparison by these standards. Even though higher face-to-image pixel proportions between 6 and 20% should not have been considered suitable to conduct a facial comparison, they were still able to produce quite high overall true positive and true negative rates in a previous study using the same sample [2]. The present study’s results may suggest that these standards could be more stringent than those currently practically required for facial comparison by morphological analysis, and further quantification research in face resolution is required.

#### Face lighting and exposure

While face resolution was a good predictor of false matches, an additional factor considered was the quality of lighting of the face, particularly focusing on under- and overexposure scenarios. The assumption is that as a face is under- or overexposed, detail is lost as pixels of the face are too dark, or too bright, to allow visibility of the underlying morphology. In addition, shadows and overexposed areas can also create artificial boundaries and overall alter the facial morphology of visible areas [11, 13]. In the present study, in isolation, under- and overexposed pixel numbers were found to not necessarily be an appropriate representation of the lighting quality as these values would be higher in higher quality images, irrespective of actual lighting quality, as the overall face image resolution was higher. The under- and overexposed pixel ratios, however, were considered a better indicator of image quality. A high UFR was found to be associated mostly with an increase in false positives, while a high OFR was associated mostly with less false positives. Mean greyscale pixel value (MGV), considered a rough measure of the overall darkness or brightness of a facial image, was aligned to the above results, with lower MGV (i.e. closer to the darkest greyscale values) associated to false positives and higher MGV associated to increased false negatives. The same relationship is highlighted by the greyscale histogram skewness, as lower skewness (i.e. darker, more evenly lit images) was significantly associated with false negative outcomes.

The fact that a high level of underexposure was related to more false positives suggests that when exposure or lighting quality of the face is low, it may lead to more incorrect outcomes, likely as a result of reducing the confidence of an analyst beyond the effects of low resolution. The resulting increase in false positives in underexposed images leads to less visible detail that could be misconstrued by an analyst as visible and increase the possibility of obtaining a false positive match, possibly due to confirmation bias inherent in humans. The opposite effect seems to occur in overexposed faces, with a decrease in exclusions of a face or matches that could be false positives. This effect suggests that when overexposed images occur, the loss of detail could be enough to dissuade an analyst from attempting a match at all (exclusion), or risking the possibility of a false positive, hence the increased number of false negatives. Overexposure was initially expected to reduce ability to identify a face as an overexposed feature would be completely lost; however, the decreased presence of false positive outcomes among the overexposed images suggests that between under- and overexposure, overexposure is preferred in facial comparison. The effects of over- and underexposure could be a factor of the specific dataset used in the present study, which included faces of Black South Africans [14] which tended to be primarily ranging between greyscale values of 70 and 90 (out of 255). Due to overall darker complexion, comparison of African faces may benefit more from brighter lighting, while darker lighting could reduce the ability to confidently visualise facial detail and increase chance of error. These are crucial concerns to be cognisant of when considering the effect of lighting on both FFC but also with facial recognition technology applications, as most of these programmes are often tested using databases including primarily individuals of White or East Asian descent [14], celebrity faces (e.g. [39]) or synthetic faces (e.g. [40]). Some of these have been retracted (e.g. [41]), or may use images from social media without consent from the individuals included (e.g. [42]).

Exposure mean was seen to reflect the outcomes of the exposure ratios, with a high exposure mean occurring in most false negative and a lower exposure mean occurring in most false positives. True outcomes were generally located in a middle range of exposure means, which may be due to the darker complexion of the subjects from the Wits face database. The greyscale histogram mean may be considered better for thresholding lighting quality than specific over- and underexposure ratios. However, this would require more extensive testing and adjustment based on skin complexion in other populations.

While no studies on the specific effects of lighting on human-based facial comparison exist, when investigated in facial recognition systems that rely on eigenface and eigenphases-based algorithms, lighting tended to have a stronger effect than other factors considered, including pixel count, blur and occlusion [43]. However, Akinbola [43] did not compute false and true match rates, but rather Euclidian distances, making direct comparison difficult. Lighting effects, however, seemed to be attenuated by image histogram equalisation [43] and may be a viable alternative to address the strong effect of lighting in facial comparison as well. Nonetheless, few courts would consider accepting artificially altered image data; hence, it might not be a viable option until tested further for a forensic setting and standardised.

Despite the promising outcomes of the quantifiable values of image quality investigated in the present study, these may not necessarily be holistic indicators of image quality. Additional factors such as contrast, sharpness, image colour clarity, distortion and blur should be further investigated as factors contributing to decreases in facial image quality. Other studies in the more technical aspects of image data capture and image artefacts from normal digital processing (e.g. [44]) demonstrate the strong effects these can have on image quality and are factors that should also be considered and investigated with multidisciplinary research including image scientists.

It was also evident, during data collection and analysis for the present study, that additional qualitative considerations, affecting image quality with potential effects for comparison, were noted. These considerations included major factors limiting optimal morphological analysis that were affecting clear visibility of certain features even in better quality images. Observed quality affecting factors were grouped based on common themes as they were not fully accounted for in the qualitative scoring described above, or in the quantitative measures captured from the images. These additional quality affecting themes included the following:

- Image distortion (primarily perspective distortion)
- Image blurriness (relating to motion blur and/or image artefacts from the digital video recorder processing innate in the CCTV recording process)
- Angle of incidence (decreased visibility of inferior components of the face)
- Lighting quality (specifically uneven lighting between parts of the face)
- Background and subject contrast (loss of edge detail due to background and subject merging)

Methods for the semi-quantitative and quantitative assessment of these additional factors should be further investigated, and a holistic approach to image quality assessment with clear thresholds for inclusion and exclusion of images should result from this. Once this is investigated in depth, developing a confidence factor based on image quality for the facial comparison outcomes could enhance defensibility of outcomes in a court setting and protect analysts when image data is of poor quality.

Regardless of long-term possibilities for this research and its applications, the authors would recommend that facial comparison analysts apply, as a minimum, the adapted image quality scoring system presented in the current study as a first trial for image triage, before actually starting the facial comparison procedure. In addition, simply importing the images into Microsoft Paint and cropping them to the closest pixel of the face, head and neck area and calculating the face-to-image pixel proportion, as described in the current study, should be performed in order to verify that it is above 6% of the overall image resolution. This specification could be used as an additional consideration of the image being of sufficient quality for facial comparison analysis. Based on these two factors, images with an image quality score of 6 should definitely be excluded from facial comparison analysis, but potentially used for facial review and investigation if required. The same would be recommended for face-to-image pixel proportions lower than 6%; however, further research would be required to identify any thresholds between 1 and 6%. As extracting the lighting information was a bit more complex and may not be feasible for all analysts, the primary recommendation from this study would be for analysts analysing faces of darker complexions to be more cognisant of the effect of underexposure in order to avoid giving in to their innate confirmation bias, and preferably select images that would be overexposed as opposed to underexposed ones that could lead to more false positives.

## Conclusions

This study has shown clear relationships between image quality factors and forensic facial comparison by morphological analysis outcomes. It has also demonstrated the importance of using a quality scoring system, such as the one adapted here from Schüler and Obertová [4], to conduct a preliminary image triage process before considering conducting a facial examination, as encouraged by the ENFSI [26]. The benefits of image triage using a scoring system are highlighted in its ability to mostly correctly predict the potential of images leading to correct matches with an accuracy of 85.9%, precision of 95.8% and recall of 83.6%. As such, being able to avoid conducting analyses with a low probability of yielding correct matches can greatly aid in both reduction of false positive and false negatives in facial comparison practice and also reduction in human resource wastage for risky analyses. Further improvements should be made to the image quality scoring system based on facial feature contribution to correct matches once that data is available. Image triage using the above quality scoring system and face-to-image pixel proportion could be easily employed by facial comparison practitioners before beginning their analysis. The lighting of faces and the likely relationship with skin complexion are important to note.

Quantification of image quality in relation to facial comparison outcomes, although promising, is a challenging endeavour and will require a large extent of fine-tuning, in collaboration with image scientists, in order to identify and consider holistically more image quality affecting factors, such as optical and perspective distortion, artefacts and image clarity. Other than investigating image quality from the image data for facial comparison, this study could also aid forensic practitioners and police services in capturing photographs of faces as well as CCTV installation companies on the ideal conditions and locations for optimal face image data capture for comparison purposes. Identifying these optimal conditions across different settings and requirements could lead to clearer instructions for CCTV installations to optimally monitor an overall area and also acquire high-quality facial images.

### Supplementary information

Below is the link to the electronic supplementary material.

ESM 1 (PDF 324 KB)

## Data Availability

The facial image data used in the current paper are part of the Wits Face Database [14, 15]; the images themselves are protected by the South African Protection of Personal Information Act and cannot be distributed without formal application and ethics approval. Sample facial images of the principal investigator are available under Creative Commons Attribution License (CC-BY). This license permits unrestricted use, distribution and re-production in any medium of the sample images, provided the original work is properly cited. The Wits Face Database data note, including the supplementary material for the Wits Face Database [14, 15], can be found at the following URL: 10.12688/f1000research.50887.1. The image quality data extracted and analysed in the current study can be made available upon request to the corresponding author. The ImageJ Macro code for image histogram data extraction can be accessed at the public code repository of github; the two macros used in the current study are the ImageJ Macro code, BatchListHistogram_General_Final.ijm, and ImageJ Macro code, BatchGrayHistogramSummaryStats_General.ijm, both publicly available at https://github.com/NBacci/Image-Analysis-ImageJ-Macros.git.
