# Supplementary material for: Prioritising quality: investigating the influence of image quality on forensic facial comparison
Source: Int J Legal Med. 2024 Feb 22;138(4):1713–26. doi: 10.1007/s00414-024-03190-7 (PMC11164719; doi:10.1007/s00414-024-03190-7)
Supplement: Supplementary file 1 — (PDF 324 KB) [file 414_2024_3190_MOESM1_ESM.pdf]

Title: Prioritizing quality: investigating the influence of image quality on forensic facial comparison

Journal: International Journal of Legal Medicine

Authors: Nicholas Bacci [1,*], Nanette Briers [2], and Maryna Steyn [3]
Affiliations:

[1] Human variation and identification research unit, School of Anatomical Sciences, Faculty of Health Sciences, University or the Witwatersrand, Johannesburg, South Africa; Nicholas.Bacci@wits.ac.za ORCID: 0000-0001-6858-7598

[2] Division of Clinical Anatomy, Faculty of Medicine and Health Sciences, University of Stellenbosch, South Africa; briers@sun.ac.za ORCID: 0000-0002-8544-6049

[3] Human variation and identification research unit, School of Anatomical Sciences, Faculty of Health Sciences, University or the Witwatersrand, Johannesburg, South Africa; Maryna.Steyn@wits.ac.za ORCID: 0000-0002-0215-9723

* Correspondence: Nicholas.Bacci@wits.ac.za; Tel.: +27 11 717 2204

Supplementary Information

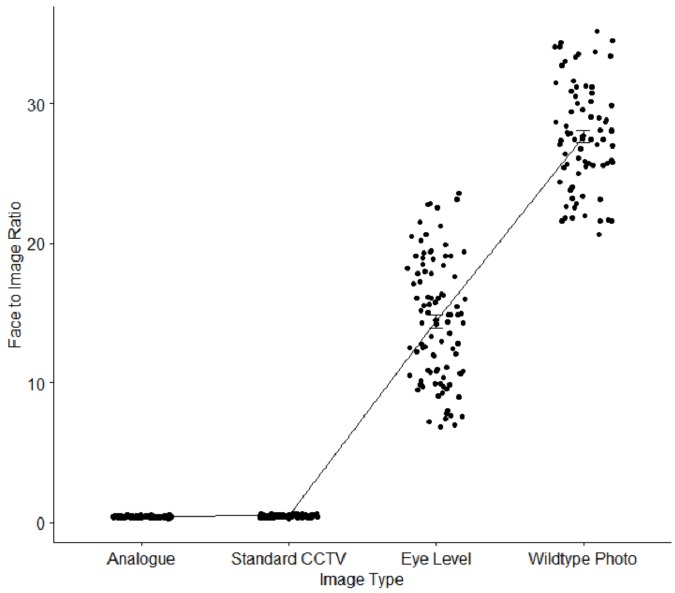

Fig. 1 Scatter plot with randomized jitter points and mean error bars of face-to-image pixel proportion by imaging modality. Significant differences are seen between all image types, each subsequent modality having a much greater number of pixels of the image that made up part of the face

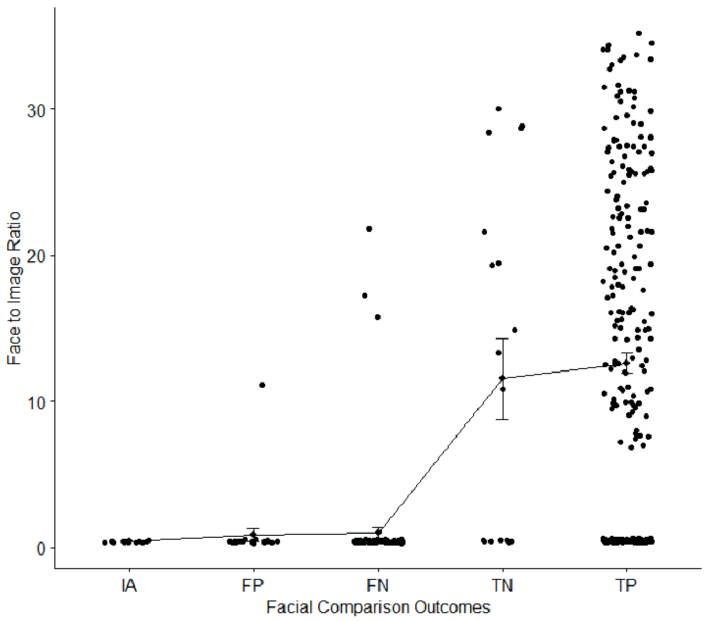

Fig. 2 Scatter plot with randomized jitter points and mean error bars of face-to-image pixel proportion by comparison outcome. Significant differences are seen between true positive (TP) and true negative (TN) outcomes, and the other three outcomes, which were generally related to lower face-to-image pixel proportion. Key: IA= Inconclusive Analysis; FP= False Positive; FN= False Negative; TN= True Negative; TP= True Positive

Table 1 Pairwise comparisons using Wilcoxon rank sum test with Bonferroni continuity correction between comparison outcomes and underexposed greyscale pixel value bins

	**False Negative**	**False Positive**	**Inconclusive Analysis**	**True Negative**
**False Positive**	< 0.001*	-	-	-
**Inconclusive analysis**	1.000	0.005*	-	-
**True Negative**	0.002*	1.000	0.045*	-
**True Positive**	< 0.001*	0.064	< 0.001*	1.000

* These values indicate significant differences between two comparison outcomes.

Table 2 Pairwise comparisons using Wilcoxon rank sum test with Bonferroni continuity correction between comparison outcomes and overexposed greyscale pixel value bins

	**False Negative**	**False Positive**	**Inconclusive Analysis**	**True Negative**
**False Positive**	1.000	-	-	-
**Inconclusive analysis**	0.718	1.000	-	-
**True Negative**	0.002*	0.020*	0.005*	-
**True Positive**	< 0.001*	< 0.001*	= 0.001*	1.000

* These values indicate significant differences between two comparison outcomes.

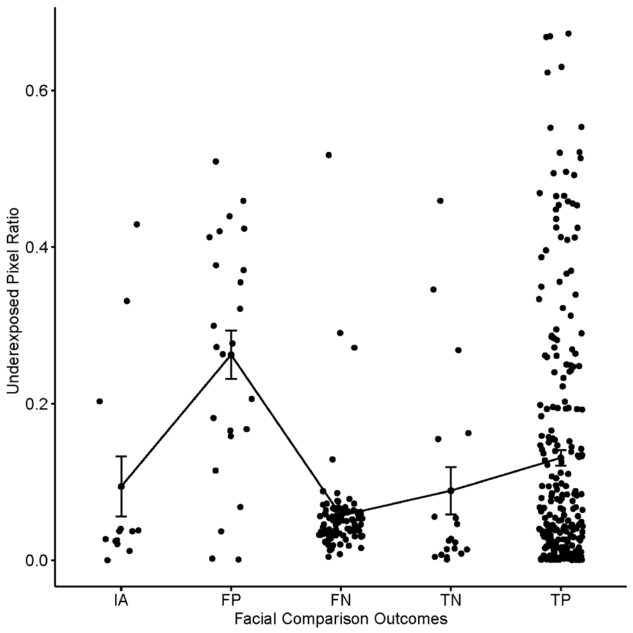

Fig. 3 Scatter plot with randomized jitter points and mean error bars of underexposed pixel proportion by comparison outcome. Significant differences are seen between false positive (FP) and all other outcomes as an increase of underexposed pixels in false positives. Key: IA= Inconclusive Analysis; FP= False Positive; FN= False Negative; TN= True Negative; TP= True Positive

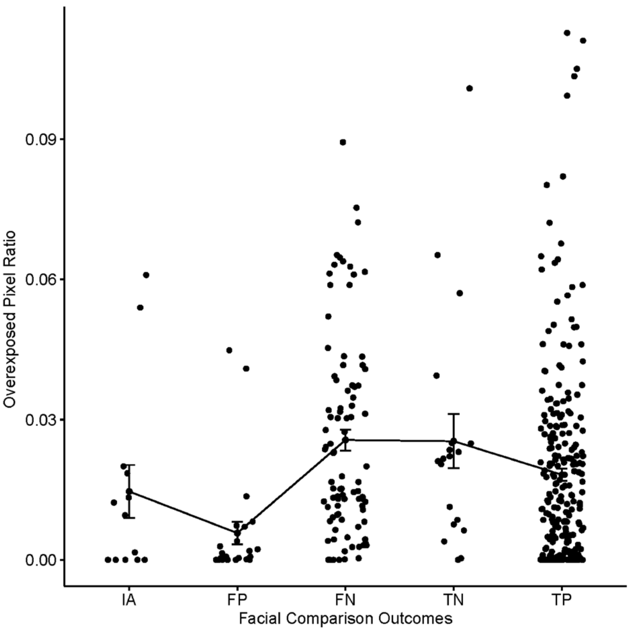

Fig. 4 Scatter plot with randomized jitter points and mean error bars of overexposed pixel ratio by comparison outcome. Significant differences are seen between false positive (FP), inconclusive analyses (IA) and true outcomes (TP and TN). This is evident with the lower ratio of overexposed pixels in FP outcomes compared to FN, TN, and TP. In addition, FP outcomes had a significantly lower ratio of overexposed pixels than TP outcomes. Key: IA= Inconclusive Analysis; FP= False Positive; FN= False Negative; TN= True Negative; TP= True Positive

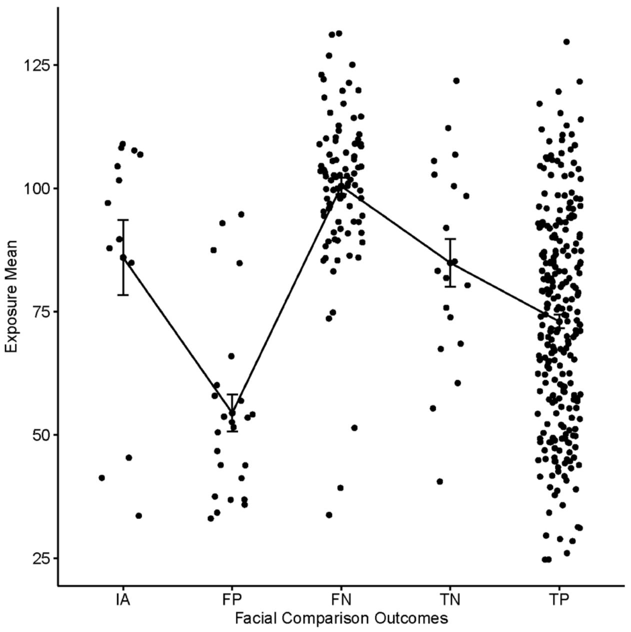

Fig. 5 Scatter plot with randomized jitter points and mean error bars of exposure mean by comparison outcome. Significant differences are seen between true positives (TP) and negative (TN), and false positives (FP) and negatives (FN), as well as between false positives and negatives. Inconclusive analyses (IA) had significantly higher mean greyscale values than false positives. FN outcomes tended to have higher mean greyscale values than FP, TN, and TP outcomes, while FP tended to have lower mean greyscale values than IA, FN, TP, and TN. Key: IA= Inconclusive Analysis; FP= False Positive; FN= False Negative; TN= True Negative; TP= True Positive

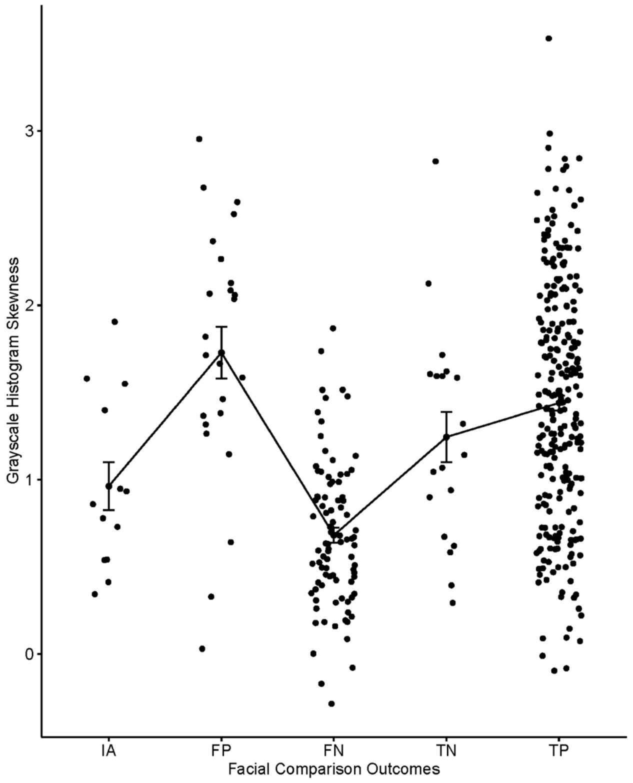

Fig. 6 Scatter plot with randomized jitter points and mean error bars of greyscale histogram skewness by comparison outcome. Significant differences are seen between false negatives (FN) and other outcomes excluding inconclusive analyses (IA). False negative outcomes were associated with a less skewed histogram, over other outcomes (excluding inconclusive analyses). Key: IA= Inconclusive Analysis; FP= False Positive; FN= False Negative; TN= True Negative; TP= True Positive
